# A Rare Case of Brucellosis With Hepatic Involvement

**DOI:** 10.7759/cureus.84631

**Published:** 2025-05-22

**Authors:** Dillon A Drab, Madeeha Javed, Shivani Desai, Colin Ly, Farhan Abdullah

**Affiliations:** 1 Internal Medicine, Methodist Dallas Medical Center, Dallas, USA

**Keywords:** brucellosis, hepatology, mexico, preventative medicine, public health

## Abstract

This case report describes a rare instance of brucellosis with hepatic involvement in a 58-year-old female who contracted the infection after ingesting contaminated dairy products from Mexico. Brucellosis is a zoonotic infection caused by gram-negative coccobacilli of the genus Brucella. The disease is relatively uncommon in the United States due to strict animal health policies; however, it remains a major public health concern in many other countries. Brucellosis typically presents with non-specific symptoms but can rapidly progress to multi-organ involvement. Clinicians should consider brucellosis in patients with non-specific symptoms, particularly those with a history of consuming potentially contaminated products. Diagnosis is typically based on cultures or serology and is subsequently treated with appropriate antibiotic therapy. Here, we present a case of brucellosis that was initially misdiagnosed due to the nonspecific nature of the presenting symptoms, which ultimately led to significant liver involvement. Culture data later confirmed Brucella, and the patient was started on a combination of doxycycline and gentamicin therapy, resulting in the resolution of the disease. This case emphasizes the need for public education on food safety and highlights the risks associated with consuming contaminated products brought into the U.S. from abroad.

## Introduction

Brucella melitensis is a facultative intracellular, gram-negative, aerobic coccobacillus that is a common cause of zoonotic infections in endemic regions. Historical accounts of diseases related to Brucella infection date back to the early 20th century [1]. The most common vectors include cattle, sheep, goats, and pigs. Endemic regions primarily include developing countries that lack strict animal vaccination programs, such as areas of Central and South America [2]. Transmission to humans occurs through the ingestion of contaminated raw or unpasteurized animal products. After ingestion, Brucella organisms survive and replicate within macrophages of the reticuloendothelial system, leading to the formation of non-caseating granulomas [3]. Initial symptoms consist of high undulant fevers, night sweats, painful lymphadenopathy, hepatomegaly, and splenomegaly. However, the disease can progress to involve nearly every organ system. Although Brucella can be directly detected in tissue or serum cultures, this method is challenging because the organism is difficult to grow on traditional media [4]. Agglutination tests allow for rapid identification, and polymerase chain reaction (PCR) techniques can now be used for confirmation [4]. Treatment depends on the severity of the infection. For uncomplicated cases presenting with gastrointestinal or flu-like symptoms, the standard regimen includes doxycycline for six weeks combined with an aminoglycoside for one to three weeks [5]. For more complicated infections such as endocarditis, neurobrucellosis, or spondylitis, antibiotic therapy must be extended for four to six months to achieve full eradication. Additionally, brucellosis can affect pregnancy, leading to preterm delivery or miscarriage [6]. While treatment is effective, several preventative measures have been implemented to avoid infection entirely. In endemic areas, avoiding the ingestion of raw or unpasteurized animal products is crucial. Thorough cooking of animal products also reduces the risk of transmission. Furthermore, educational programs in these regions provide resources for early disease recognition and prevention [7]. For animal eradication efforts, vaccines are administered to livestock to prevent Brucella infection [8]. This case was previously presented at the Methodist Dallas GME Research event in February 2025.

## Case presentation

A 58-year-old female with no known past medical history initially presented with fever, lower quadrant abdominal pain, and malaise ongoing for one week. Initial vital signs revealed a mild fever and tachycardia, along with leukocytosis. Physical examination was notable for abdominal tenderness in the lower quadrants. Further workup revealed leukocytes in the urine. Additionally, she had mildly elevated liver enzymes, including aspartate transferase (AST) at 83 U/L, alanine transaminase (ALT) at 57 U/L, and alkaline phosphatase (ALP) at 196 U/L (Table 1). Abdominal imaging at the time showed no acute abnormalities but did reveal mild hepatic steatosis. She was diagnosed with a urinary tract infection based on urinalysis findings alone and discharged with a prescription for cephalexin. Initial cultures showed no growth. The elevated liver enzymes were attributed to hepatic steatosis as visualized on imaging. However, after five days, blood cultures grew Brucella, later speciated as Brucella melitensis. The patient was notified and instructed to return for further evaluation and treatment. On her second presentation, her symptoms had progressed, with the development of nausea, vomiting, and poor appetite. Blood cultures were re-drawn and again grew Brucella. During this admission, liver enzymes rose significantly, with AST at 308 U/L, ALT at 174 U/L, and ALP at 322 U/L (Table 2). Further hepatic workup confirmed hepatic steatosis but was negative for viral hepatitis and autoimmune causes. The acute elevation in liver enzymes was thought to be secondary to brucellosis. She was initiated on doxycycline and gentamicin, which led to improvement in both liver enzymes and clinical symptoms. Given the downward trend in liver enzyme levels, liver biopsy was deferred, with plans for routine surveillance as the patient continued antibiotic treatment. After an extensive discussion with the patient and her family, it was revealed that a recent visiting family member from Mexico had experienced similar symptoms. This family member had also brought dairy products from Mexico, identifying these as the likely source of the infection due to potential contamination.

**Table 1:** Initial liver enzymes Initial liver enzymes during the first presentation. AST, aspartate transferase; ALT, alanine transaminase; ALP, alkaline phosphatase

	Value (U/L)	Reference Range (U/L)
AST	83	9-50
ALT	57	5-50
ALP	196	40-125

**Table 2:** Secondary hospital presentation Liver enzymes on the second presentation. AST, aspartate transferase; ALT, alanine transaminase; ALP, alkaline phosphatase

	Value (U/L)	Reference Range (U/L)
AST	308	9-50
ALT	174	5-50
ALP	322	40-125

## Discussion

Brucellosis remains a public health concern in endemic regions but is highly treatable and associated with low mortality rates [2]. Given the continued importation of agricultural products from endemic areas, brucellosis should remain an important consideration for clinicians practicing in regions with at-risk populations. This case highlights the diagnostic challenge of brucellosis. In its early stages, symptoms are often non-specific and may include undulant fevers, night sweats, painful lymphadenopathy, hepatomegaly, and splenomegaly. Routine laboratory studies in the initial phases may reveal anemia, thrombocytopenia, elevated creatine kinase, and mildly elevated transaminases [4]. As in the case presented, the patient's symptoms were initially attributed to a urinary tract infection, and only a short course of inappropriate antibiotics was administered. Had blood cultures not been performed, the patient might have developed a more severe infection, potentially progressing to fulminant liver failure. Other serious complications of Brucella spp. infection include neurobrucellosis - manifesting as meningitis, encephalitis, or optic neuritis - endocarditis, orchitis (which may result in sterility), and osteomyelitis [2]. Once infections advance to these stages, extended courses of combination antibiotic therapy are required for complete eradication. Common regimens include combinations of cephalosporins, tetracyclines, or rifamycins [9].

This case also underscores the challenges in isolating Brucella spp. The organism requires specialized media, such as charcoal yeast agar buffered with cysteine and iron, to support growth [10]. Blood cultures may take seven to 14 days to yield results, and false negatives are relatively common (4). Often, repeat sampling is necessary to increase the likelihood of detection, contributing to delays in diagnosis and treatment.

Preventive efforts have been successful in most developed countries. For example, Brucella vaccines are routinely administered to commercial calves to reduce infection rates. In the United States, cattle herds are tested at least once annually, and infected animals are typically euthanized to prevent disease spread [8]. Furthermore, educating at-risk individuals is critical in reducing transmission. Preventive measures include avoiding the consumption of raw or unpasteurized animal products, likely the source of infection in the present case.

## Conclusions

While Brucella is common worldwide, it is rare in the United States due to current animal health policies. The initial presentation is often non-specific, with generalized symptoms; however, if not promptly treated, the infection can progress to involve other organs, as demonstrated in this case with hepatic involvement. In general, treatment success is high and mortality is low when appropriate antibiotics are administered. Ongoing eradication efforts, combined with public education, are essential to preventing future outbreaks.
